# Complete genome of *Pseudomonas chlororaphis* strain UFB2, a soil bacterium with antibacterial activity against bacterial canker pathogen of tomato

**DOI:** 10.1186/s40793-015-0106-x

**Published:** 2015-12-01

**Authors:** Peng Deng, Xiaoqiang Wang, Sonya M. Baird, Shi-En Lu

**Affiliations:** Department of Biochemistry, Molecular Biology, Entomology and Plant Pathology, Mississippi State University, Mississippi State, USA; Department of Plant Pathology, Shandong Agricultural University, Taian, 271018 Shandong China

**Keywords:** *Pseudomonas chlororaphis* strain UFB2, Complete genome, Biocontrol, Bacterial canker of tomato, Secondary metabolites

## Abstract

Strain UFB2 was isolated from a soybean field soil in Mississippi and identified as a member of *Pseudomonas chlororaphis*. Strain UFB2 has a broad-spectrum antimicrobial activity against common soil-borne pathogens. Plate assays showed that strain UFB2 was especially efficient in inhibiting the growth of *Clavibacter michiganensis* 1–07, the causal agent of the devastating bacterial canker of tomato. Here, the complete genome sequence of *P. chlororaphis* strain UFB2 is reported and described. The strain UFB2 genome consists of a circular chromosome of 6,360,256 bp of which 87.86 % are protein-coding bases. Genome analysis revealed multiple gene islands encoding various secondary metabolites such as 2,4-diacetylphloroglucinol. Further genome analysis will provide more details about strain UFB2 antibacterial activities mechanisms and the use of this strain as a potential biocontrol agent.

## Introduction

Bacterial strains of *Pseudomonas chlororaphis* are aerobic Gram-positive bacteria and many of the strains possess a wide-spectrum antifungal activity against soil-borne plant pathogens [1–5]. *P. chlororaphis* strains have been reported to be efficient plant-growth-promoting bacteria, which can be used as inoculants for biofertilization, phytostimulation and biocontrol [6]. The use of *P. chlororaphis* strains as biocontrol agents is promising because they are capable of producing a variety of antimicrobial secondary metabolites including phenazine-1-carboxamide, 2-hydroxyphenazine, pyrrolnitrin, hydrogen cyanide, chitinases and proteases [6–8]. Moreover, *P. chlororaphis* is considered to be nonpathogenic to humans, wildlife, or the environment according to U.S. environmental protection agency (EPA) [9]. Antimicrobial activities and low risks to animals and the environments have made the bacterium *P. chlororaphis* highly potential biocontrol agents in agriculture [8, 10]. A genome-wide research and analysis could provide useful information about the mechanisms of how *P. chlororaphis* protects plants against soil-borne phytopathogens. Currently, the whole genomes of a few *P. chlororaphis* strains that exhibit antifungal activity have been sequenced. These include *P. chlororaphis* strain PA23 that can protect canola from stem rot disease caused by the fungal pathogen *Sclerotinia sclerotiorum* [2, 11], and *P. chlororaphis* PCL1606 that was isolated from avocado rhizosphere and exhibited biocontrol activity against soil-borne phytopathogenic fungi [1]. In addition, another functionally-uncharacterized strain, *P. chlororaphis* subsp. *aurantiaca* JD37, was recently sequenced (NCBI reference sequence: NZ_CP009290.1). Genome sequences of *P. chlororaphis* strains with significant antibacterial activity have not been reported previously.

Strain UFB2 was isolated from a soybean field soil in Mississippi. Preliminary analysis of the 16S rRNA gene indicated that it is a member of *P. chlororaphis*. Plate assays indicated *P. chlororaphis* strain UFB2 has a broad spectrum of antimicrobial activities, especially against bacterial canker pathogen of tomato: *Clavibacter michiganensis* [12, 13]. Greenhouse trials demonstrated both living cells and culture extract of strain UFB2 can be used for disease management of bacterial canker of tomato. In this study, the *P. chlororaphis* strain UFB2 complete genome sequence and annotation are reported. The gene islands within strain UFB2 genome that encode various secondary metabolites, including antimicrobial compounds, are also described. The detailed description of the strain UFB2 genome will shed light into further studies of biocontrol effectiveness and applications of *Pseudomonas* species.

## Organism information

### Classification and features

**Fig. 1:**
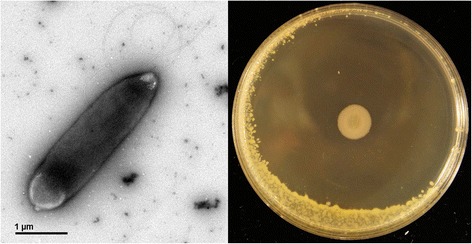
Image of *P. chlororaphis* UFB2 cells and plate assay of UFB2 antibacterial activity against *Clavibacter michiganensis* 1*–*07. The plate bioassay was conducted as described by Scholz-Schroeder and colleagues [44]

**Fig. 2:**
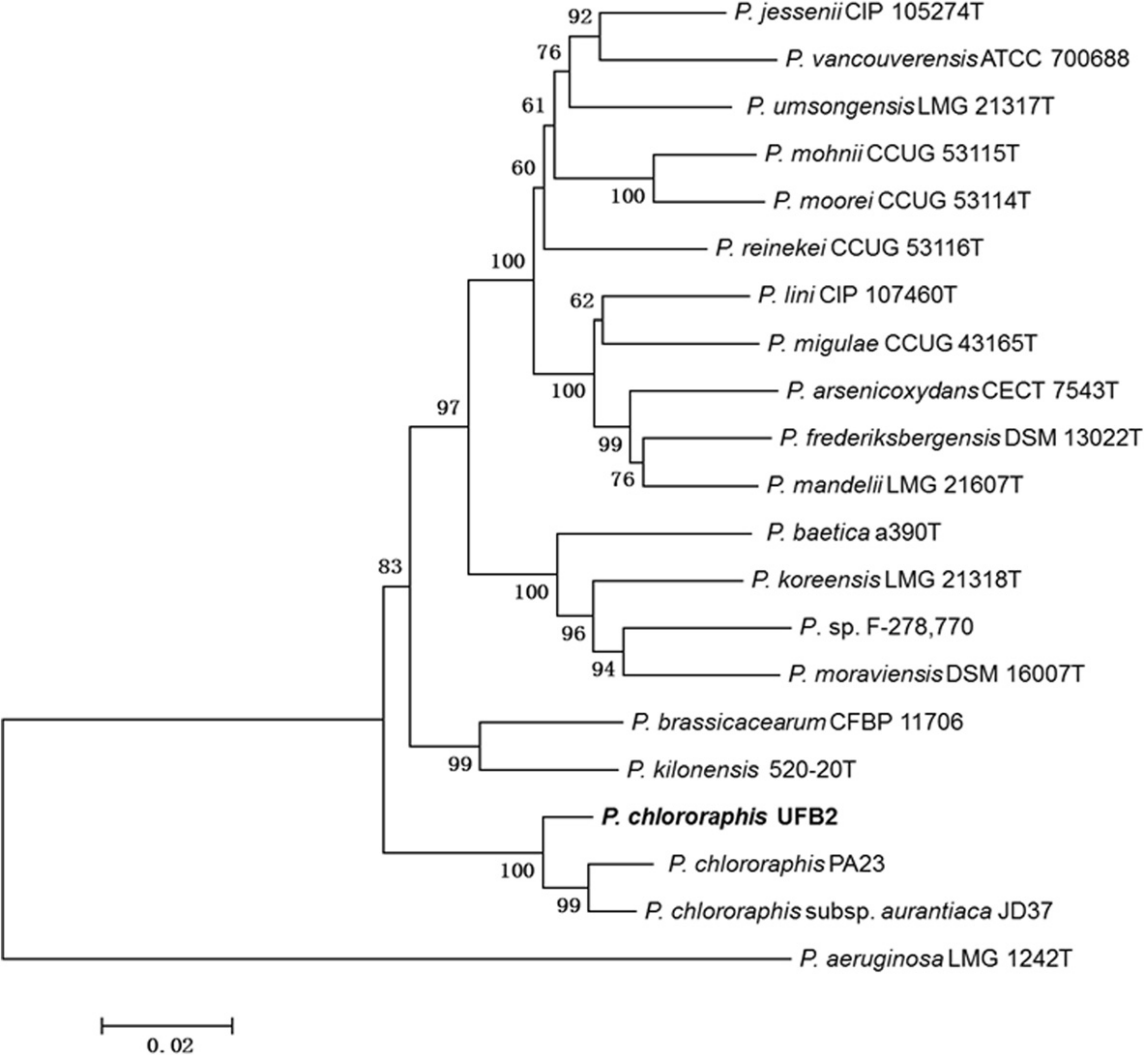
Phylogenetic analysis of concatenated four multilocus sequence typing loci of *P. chlororaphis* UFB2 and related species. Phylogenetic tree based on the concatenated sequence (3775 bp) of four housekeeping gene fragments [*gyrB* (729 bp), *rpoB* (885 bp), *rpoD* (711 bp) and 16 s rRNA (1450 bp)]. Phylogenetic analyses were performed using MEGA, version 6.06 [51]. The tree was built using the Neighbor-Joining method [52]. Bootstrap analysis with 1000 replicates was performed to assess the support of the clusters

Strain UFB2 was isolated from rhizosphere soil sample collected from soybean field near Cleveland, Mississippi, USA, where healthy soybean plants were found growing in charcoal rot disease patch. Phylogenetic analyses based on multilocus sequence typing [14] (gyrB, rpoB, rpoD and 16 s rRNA) revealed that strain UFB2 belongs to *Pseudomonas chlororaphis* (Fig. 2). Strain UFB2 is rod-shaped, motile, non-spore-forming Gram-negative bacterium in the order *Pseudomonadales* of the class *Gammaproteobacteria*. UFB2 cells are approximately 3.0 ± 0.8 μm in width and 0.9 ± 0.3 μm in length (Fig. 1). The strain is relatively fast-growing, forming approximately 1 mm opaque yellowish colonies after overnight incubation at 28 °C on nutrient-broth yeast extract agar [15]. Strain UFB2 can also be grown on rich media such as LB [16] and PDA, as well as M9 minimal medium [17]. Phenotypic characterization of strain UFB2 was carried out using the API 50CH system as recommended by manufacturer. According to the result, strain UFB2 could utilize almost all carbon sources in API 50CH tests, including D-glucose, D-galactose, L-rhamnose, D-mannitol, D-raffinose, D-fructose, D-arabinose, D-ribose, L-arabinose, L-xylose and D-xylose, but not potassium gluconate.

Plate bioassays demonstrated that strain UFB2 possesses significant antibacterial activity against a broad array of plant bacterial pathogens. Other than *Clavibacter michiganensis* 1–07, the tested bacteria sensitive to strain UFB2 also include *Erwinia amylovora* [18, 19], *Burkholderia glumae* [20], *Ralstonia solanacearum* Rso [21, 22] and *Erwinia carotovora*WSCH1 [19, 23]. Of the tested plant pathogenic bacteria, the Gram-positive bacterium *Clavibacter michiganensis* 1–07*,* the pathogen causing bacterial canker of tomato [24], is most sensitive to strain UFB2 with a radius of 28 ± 1 mm clear inhibitory zone (Fig. 1). In addition, the growth of fungal pathogen *Geotrichum candidum* Km, which causes sour rot of citrus fruits, tomatoes, carrot and some vegetables [25], can also be inhibited by strain UFB2. To test the field biocontrol efficacy of strain UFB2, greenhouse experiments were set up according to the method described by Lu and Ingram [26]. Preliminary data showed the control efficacies of both strain UFB2 culture extract and living cells on bacterial canker of tomato are equivalent to that of streptomycin at the recommended rate for plant disease management. The genome of strain UFB2 was sequenced with the aim to identify the genes associated with the antimicrobial characters. The information about the genome sequence of strain UFB2 is summarized in Table 1, and its phylogenetic position is shown in Fig. 2.

**Table 1:** Classification and general features of *Pseudomonas chlororaphis* UFB2 according to the MIGS recommendations [55]

MIGS ID	Property	Term	Evidence code^a^
	Classification	Domain *Bacteria*	TAS [56]
		Phylum *Proteobacteria*	TAS [57]
		Class *Gammaproteobacteria*	TAS [58, 59]
		Order *Pseudomonadales*	TAS [19, 60]
		Family *Pseudomonadaceae*	TAS [19, 61]
		Genus *Pseudomonas*	TAS [19, 61–63]
		Species *Pseudomonas chlororaphis*	TAS [19, 64, 65]
		strain: *UFB2*	NAS
	Gram stain	negative	TAS [66]
	Cell shape	Rod	TAS [66]
	Motility	Motile	TAS [66]
	Sporulation	None	NAS
	Temperature range	Mesophilic	IDA
	Optimum temperature	33 °C	IDA
	pH range; Optimum	not determined	IDA
	Carbon source	D-glucose, D-galactose, L-rhamnose, D-mannitol, D-raffinose, D-fructose, D-arabinose, 2D-ribose, L-arabinose, L-xylose, D-xylose.	TAS [66]
MIGS-6	Habitat	Soil	NAS
MIGS-6.3	Salinity	not determined	IDA
MIGS-22	Oxygen requirement	Aerobic	NAS
MIGS-15	Biotic relationship	free-living/Rhizospheric	NAS
MIGS-14	Pathogenicity	non-pathogen	IDA
MIGS-4	Geographic location	Mississippi, USA	IDA
MIGS-5	Sample collection	2011	IDA
MIGS-4.1	Latitude	34.1 N	IDA
MIGS-4.2	Longitude	90.6 W	IDA
MIGS-4.4	Altitude	40 M	IDA

^a^Evidence codes - *IDA* Inferred from Direct Assay, *TAS* Traceable Author Statement (i.e., a direct report exists in the literature), *NAS* Non-traceable Author Statement (i.e., not directly observed for the living, isolated sample, but based on a generally accepted property for the species, or anecdotal evidence). These evidence codes are from the Gene Ontology project [67]

#### Chemotaxonomic data

Fatty acid analysis was performed by gas chromatography (gas chromatograph, model 6890 N, Agilent Technologies) and analyzed by the Microbial Identification System (MIDI, Sherlock Version 6.1; database, TSBA40). The analysis of total cells showed the major fatty acids are C _16:1_^ω7c^ (32 %), C _16:0_ (28 %), C _18:1_^ω7c^ (19 %). Fatty acid 3-hydroxy C _12:0_ (5 %), C _12:0_ (4 %), 2-hydroxy C _12:0_ (4 %) and 3-hydroxy C _10:0_ (3 %) were found in minor amount.

## Genome sequencing information

### Genome project history

*P. chlororaphis* strain UFB2 was selected for sequencing because of its significant antimicrobial activities and its potential as a biocontrol agent for agricultural use. Genomes of three *P chlororaphis* strains have been sequenced as of May 2015. Sequencing of the whole genome of strain UFB2 makes more data available for genome comparison and analysis within *P. chlororaphis* species.

The genome project is deposited in the Genomes OnLine Database [27] and the NCBI BioProject database [28]. The annotated genome is publicly available from the Intergrated Microbial Genomes Database [29] under the accession number Gp0111981 and GenBank under accession number CP011020. A summary of the project information is provided in Table 2.

**Table 2:** Project information

MIGS ID	Property	Term
MIGS 31	Finishing quality	Finished
MIGS-28	Libraries used	libraries of 400 bp, mate pair library of 2,000, 5,000 and 8,000 bp
MIGS 29	Sequencing platforms	Illumina
MIGS 31.2	Fold coverage	600 ×
MIGS 30	Assemblers	DNAStar Seqman NGen v12
MIGS 32	Gene calling method	NCBI Prokaryotic Genome Annotation Pipeline
	Locus Tag	VM99
	Genbank ID	CP011020
	GenBank Date of Release	Jun 9^th^, 2015
	GOLD ID	Gp0111981
	BIOPROJECT	PRJNA277727
MIGS13	Source Material Identifier	UFB2
	Project relevance	Biocontrol

### Growth conditions and genomic DNA preparation

*P. chlororaphis* strain UFB2 was cultured in liquid NBY medium overnight at 28 °C in a shaker at 220 rpm. The genomic DNA was extracted from 50 mL of the culture using the Wizard Genomic DNA Purification Kit (Promega Corporation, Madison, WI, USA). Totally approximately 900 μg of DNA were obtained with an OD260/280 of 1.9. The DNA sample was used for library construction with Illumina Genomic DNA Sample Preparation Kit (Illumina, CA, USA).

### Genome sequencing and assembly

One standard library with an average insert size of 400 bp and three mate pair libraries with an average insert size of 2,000 bp, 5,000 bp and 8,000 bp were prepared and sequenced on the Illumina MiSeq instrument according to the manufacturer’s instructions. The genome was *de novo* assembled using a method as described by Durfee et al. [30] using DNAStar Seqman NGen (Version 12, DNASTAR, Inc. Madison, WI U.S.). The standard library and 2,000 bp mate pair library were selected for the *de novo* assembly. A total of 30 million short reads were scanned and extracted from the raw data files as input data. The short reads were preprocessed by Seqman NGen to trim adaptors and filter low-quality reads. Automatic Mer size and a minimum match percentage of 98 % were selected. 29 million short reads were assembled into 29 contigs and SeqMan Pro (Version 12, DNASTAR, Inc. Madison, WI U.S.) was used to order the contigs in one scaffold according to the mate pair data. The first round assembled sequence was then used as a template for a complete reassembly. The 2,000 bp and 8,000 bp mate pair data were incorporated to proofread the first assembly and to maximize coverage and quality. Adjacent contigs, if possible, were merged. Remaining gaps were filled by PCR and Sanger sequencing. No contigs that might correspond to plasmids remained unassembled. IslandViewer [31] was used to predict and identify genomic islands.

### Genome annotation

Automatic annotation was performed using the NCBI Prokaryotic Genome Annotation Pipeline [32], which combines gene calling algorithm with similarity-based gene detection approach to predict protein-coding genes, structural RNAs (5S, 16S, 23S), tRNAs and small non-coding RNAs. Additional gene prediction analysis and functional annotation were performed by the Integrated Microbial Genomes platform [29].

## Genome properties

**Fig. 3:**
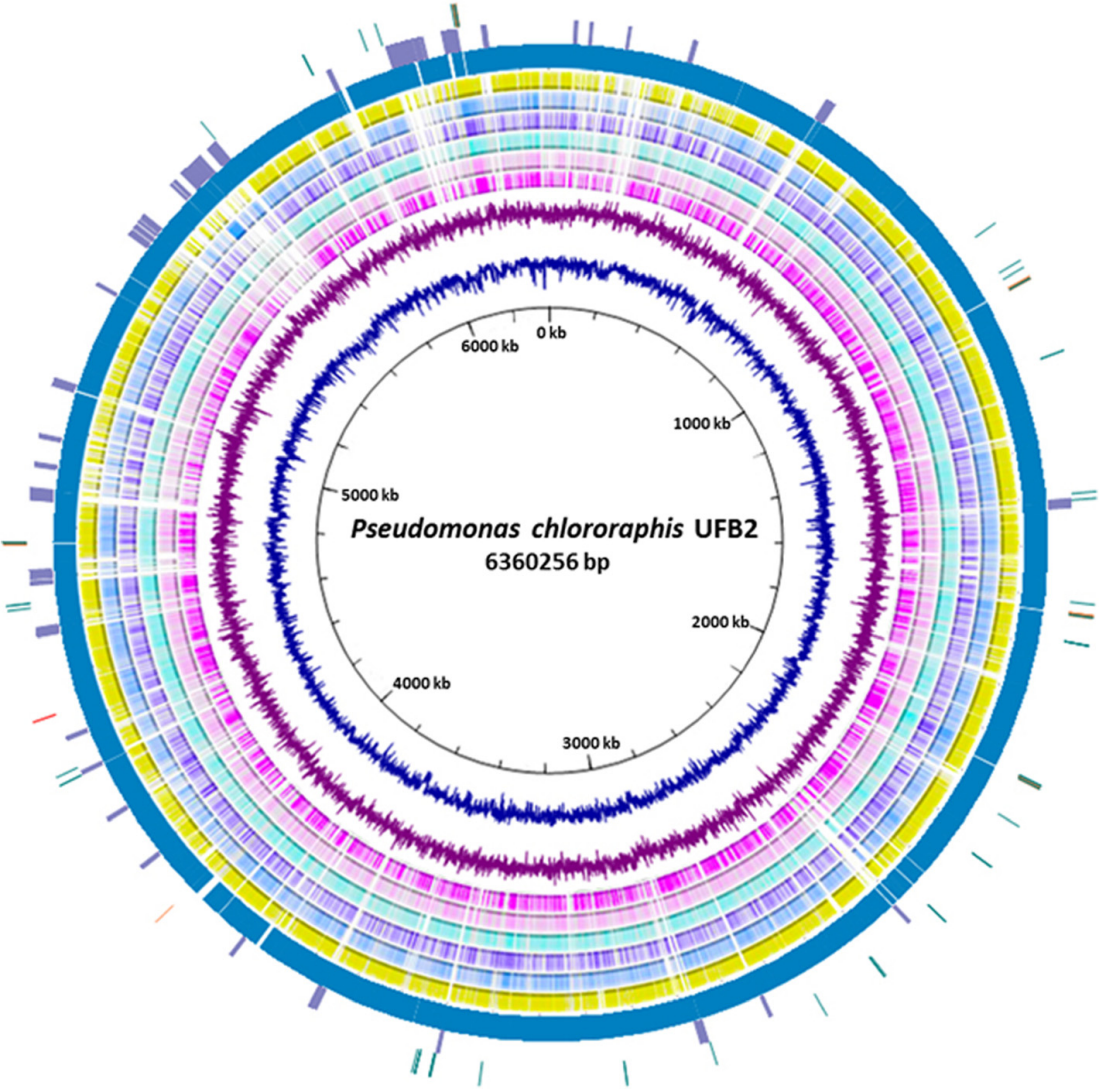
Circular representation of the *P. chlororaphis* UFB2 genome compared with six sequenced *Pseudomonas* whole genomes. Rings from inside to outside: (1) Scale, (2) GC content (navy), (3) GC skew (purple), (4) BLAST comparison with *P. syringae* pv. *syringae* B728a (deep pink), (5) BLAST comparison with *P. putida* KT2440 (pink), (6) BLAST comparison with *P. chlororaphis* strain PA23 (cyan), (7) BLAST comparison with *P. aeruginosa* PAO1 (violet), (8) BLAST comparison with *P. fluorescens* Pf0-1 (skyblue), (9) BLAST comparison with *P.* sp. UW4 (yellow), (10) Coding sequences of *P. chlororaphis* UFB2 genome (dark cyan), (11) Gene islands (medium purple), (12) rRNA genes (orange), tRNA genes (dark green) and ncRNA (red). BLASTn comparison of genomes was visualized by BRIG [53] and UFB2 genome the image was generated with Circos [54]

The complete genome of *P. chlororaphis* strain UFB2 consists of one circular chromosome of 6,360,256 bp with a GC content of 62.03 %. 5,556 genes were identified from the genome, of which 5,473 are protein coding genes. 90 of the 5,556 genes were predicted to be pseudogenes or partial genes. The genome encodes 1 noncoding RNA, 5 rRNA operons and 65 tRNAs. Seventy genomic islands ranging from 4 kbp to 43.5 kbp were also identified throughout the strain UFB2 genome, among which majority of the islands encode hypothetical proteins. The genome features of *P. chlororaphis* strain UFB2 are summarized in Tables 3 and 4, and the circular chromosome of strain UFB2 is shown in Fig. 3.

**Table 3:** Genome statistics

Attribute	Value	% of Total
Genome size (bp)	6,360,256	100.00
DNA coding (bp)	5,588,126	87.86
DNA G + C (bp)	3,945,558	62.03
DNA scaffolds	1	100.00
Total genes	5,556	100.00
Protein coding genes	5,473	98.51
RNA genes	83	1.49
Pseudo genes	90	1.62
Genes in internal clusters	5,473	98.51
Genes with function prediction	4,886	87.94
Genes assigned to COGs	4,092	73.65
Genes with Pfam domains	4,748	85.46
Genes with signal peptides	577	10.39
Genes with transmembrane helices	1,228	22.10
CRISPR repeats	0	0

**Table 4:** Number of genes associated with general COG functional categories

Code	Value	% age	Description
J	231	4.89	Translation, ribosomal structure and biogenesis
A	1	0.02	RNA processing and modification
K	418	8.85	Transcription
L	123	2.60	Replication, recombination and repair
B	3	0.06	Chromatin structure and dynamics
D	39	0.83	Cell cycle control, Cell division, chromosome partitioning
V	101	2.14	Defense mechanisms
T	316	6.69	Signal transduction mechanisms
M	262	5.55	Cell wall/membrane biogenesis
N	166	3.52	Cell motility
W	44	0.93	Extracellular structures
U	137	2.90	Intracellular trafficking and secretion
O	166	3.52	Posttranslational modification, protein turnover, chaperones
C	304	6.44	Energy production and conversion
G	227	4.81	Carbohydrate transport and metabolism
E	483	10.23	Amino acid transport and metabolism
F	92	1.95	Nucleotide transport and metabolism
H	242	5.12	Coenzyme transport and metabolism
I	234	4.96	Lipid transport and metabolism
P	257	5.44	Inorganic ion transport and metabolism
Q	142	3.01	Secondary metabolites biosynthesis, transport and catabolism
R	430	9.11	General function prediction only
S	260	5.51	Function unknown
-	1464	26.35	Not in COGs

The total is based on the total number of protein coding genes in the genome

## Insights from the genome sequence

Blast research of *P. chlororaphis* strain UFB2 genome against *P. syringae* pv. *syringae* B728a (NC_007005), *P. putida* KT2440 (NC_002947), *P. chlororaphis* strain PA23 (NZ_CP008696), *P. aeruginosa* PAO1 (NC_002516), *P. fluorescens* Pf0-1 (NC_007492) and *P*. sp. UW4 (NC_019670) genome revealed multiple unique gene regions which were only found in the strain UFB2 genome (Fig. 3). The BLASTn atlas showed noticeable genome diversity of strain UFB2 when compared to other *Pseudomonas* species. Seventy genomic islands ranging from 4 kbp to 30 kbp were also identified throughout the strain UFB2 genome, indicating significant horizontal gene transfers occurred during the evolution of strain UFB2 to better adapt the environment it inhabited.

*P. chlororaphis* strain UFB2 harbors an intact *phl* gene cluster (VM99_23970-23995), which is responsible for biosynthesis of the antimicrobial compound 2,4-diacetylphloroglucinol [33, 34]. 2,4-diacetylphloroglucinol is an especially efficient agent against soil borne fungal plant pathogens [35]. The *phl* gene cluster is involved in the *Pseudomonas* antifungal activity against *Clavibacter michiganensis* 1–07 [36]. Hydrogen cyanide [37, 38] biosynthesis gene homologs were also identified in strain UFB2 genome. The production of hydrogen cyanide by *Pseudomonas* species helps protect plants from soil-borne fungal pathogens [39, 40]. Biosynthetic gene clusters of common *Pseudomonas* species-produced antibiotics such as phenazine [41], pyrrolnitrin [42] and pyoluteorin [43] were not identified in strain UFB2 genome. Biosynthetic gene clusters of common toxins that contribute to plant and animal pathogenicity and/or virulence of *Pseudomonas* species were also searched for within strain UFB2 genome. The toxin biosynthetic gene cluster that were not identified in strain UFB2 genome include the phytotoxin lipopeptide syringomycin [44], tobacco wildfire spotting causal agent tabtoxin [45], bacterial canker of kiwifruit causal agent phaseolotoxin [46], plant-hormone-mimic toxin coronatine [47], and cytotoxic agent pederin [48]. Strain UFB2 genome harbors homolog genes to those in the bacterial apical necrosis causal agent mangotoxin [49] biosynthesis gene cluster. However, *mboC* gene homolog that is required for mangotoxin production [50] was not identified in strain UFB2 genome. Overall, the lack of the key pathogenicity/virulence genes in strain UFB2 further indicates that strain UFB2 has a great potential as a biocontrol agent.

## Conclusions

The complete genome sequence of *P. chlororaphis* strain UFB2 is described in this report. The strain UFB2 was originally isolated from the rhizosphere of a healthy soybean plant growing in a group of plants exhibiting charcoal rot disease in Mississippi. This strain possesses significant antimicrobial activities against a wide range of plant pathogenic bacteria and fungi. It is evident that the genome of *P. chlororaphis* strain UFB2 harbors the complete gene set for production of the antimicrobial compounds 2,4-DAPG and HCN, which may largely contribute to its antimicrobial activities. However, gene homologs required for biosynthesis of all the known toxins to plants, such as syringomycin, tabtoxin, phaseolotoxin, tolaasin, coronatine, or pederin, were absent from the strain UFB2 genome. The genome sequence of *P. chlororaphis* strain UFB2 will help in understanding genetic mechanisms of the antimicrobial activity studies that are useful for development of biologically-based disease management in agriculture.
